# The Current Status of European and National Financial Sources for Clinical Research and Their Impact on Paediatric Non-commercial Clinical Trials: A Case Study of the Czech Republic

**DOI:** 10.1007/s43441-020-00173-9

**Published:** 2020-06-05

**Authors:** L. Horavova, K. Nebeska, L. Souckova, R. Demlova, P. Babula

**Affiliations:** 1 Department of Applied Pharmacy, Faculty of Pharmacy, University of Veterinary and Pharmaceutical Sciences Brno, Brno, Czech Republic; 2 European Clinical Research Infrastructure Network (ECRIN), Paris, France; 3 Department of Pharmacology, Faculty of Medicine, Masaryk University, Kamenice 753/5, 625 00 Brno, Czech Republic; 4 University Hospital St. Anne’s Brno – International Clinical Research Center, Brno, Czech Republic; 5 Department of Clinical Trials, Masaryk Memorial Cancer Institute Brno, Brno, Czech Republic; 6 Department of Physiology, Faculty of Medicine, Masaryk University, Brno, Czech Republic

**Keywords:** Non-commercial Clinical Trials, Paediatric clinical research, Financial sources, International Clinical Trials Registries, Infrastructure

## Abstract

**Introduction:**

Paediatric non-commercial interventional clinical trials (NICTs) are crucial for healthcare provision. In spite of the fact that current regulations and initiatives try to enhance the quantity and quality of paediatric NICTs, there are still shortcomings that need to be addressed in order to accelerate the conduct of relevant clinical trials in children. To improve the current landscape of paediatric clinical research, it is necessary to identify and analyse the main trends and shortcomings, along with their impact on national performance in paediatric NICTs and this is the aim of this work.

**Method:**

A retrospective systematic search of paediatric NICTs was performed on four international clinical trials registries. Entries were filtered by date from 01/01/2004 to 31/12/2017. Each identified paediatric NICT was screened and analysed for sponsors, funders, type of intervention, therapeutic area, design characteristics and associated publications.

**Results:**

The search identified 439 unique NICTs. When stratifying the trials by enrolment ages, 86 trials were found involving the paediatric population. Most trials investigated the use of medicinal products and were focused on cancer or cardiovascular diseases. The most common sources of the funding were non-profit organizations. Furthermore, from the total number of completed trials, only half of them already published their results.

**Conclusion:**

The main shortcomings—specifically, ethical, methodological and, in particular, economic obstacles were identified. There is a continual need for greater support and collaboration between all major stakeholders including health policymakers, grant agencies, research institutions, pharmaceutical industries and healthcare providers at the national and international level.

**Electronic supplementary material:**

The online version of this article (10.1007/s43441-020-00173-9) contains supplementary material, which is available to authorized users.

## Introduction

Although children are our future and they should have the same options and conditions of treatment as adult patients, it is not the current reality [1]. The most common situation in paediatrics is treatment using medical products authorized for adults with an adjustment of dose or drug dosage form [2]. The majority of drugs for children are prescribed empirically, based on physician’s experiences on an “off-label” regime and the authorization for children is missing [3]. In comparison with adult medicine, it is striking because adult patients are treated exclusively with drugs developed, authorized and confirmed in clinical trials designed for the adult population. Herein lies the challenge, a child is not just a small adult and in the absence of specific trial-based data on children, clinicians are forced to extrapolate from results of trials on adults [4]. This extrapolation is often inappropriate because children have a different range of diseases and the drugs hold different pharmacokinetic and pharmacodynamic characteristics in children, resulting in responses to treatment that are unpredictably different to adults and can be endangering [5]. The development of new drugs specially for children is rare, and therefore, there is no choice but to use adult medicines for children; still, these drugs should have their viability confirmed in paediatric clinical trials [6]. However, paediatric clinical trials have been largely neglected over the long term which has led to a lack of available data of efficacy and safety even of drugs which we have been administering for decades [7].

The off-label use of adult medicines is a result of the fact that conducting paediatric clinical trials is more challenging than those conducted on adults [8]. The pharmaceutical companies are not interested in conducting clinical trials on the paediatric population [9]. For pharmaceutical companies, clinical trials are a basic tool to get authorization and approval for marketing of their new medicinal products [10]. The available market of medicines for children is of a much lower quantity and quality as for the adult population [11]. The conducting of costly and time-consuming paediatric clinical trials tends to be pointless for pharmaceutical companies [12]. It will not increase their sales through more prescribed drugs because they are already prescribed in an “off-label” regime [13]. The non-refundable costs embedded in paediatric clinical trials represent an investment that many companies cannot afford nowadays [14].

The regulatory authorities throughout Europe react to this to create smoother conditions and supporting initiatives for conducting paediatric clinical trials. To address the paucity of paediatric research, many regulations have been launched with the primary objective being a shift in mindset from “protecting children from research” to “protecting children through research” [15]. In practice, the development of medicinal products for the paediatric population within Europe is now obligatory. For instance, the European Paediatric Regulation requires applications for marketing authorizations to be accompanied by either a product-specific waiver or a paediatric investigation plan, to be approved by the European Medicines Agency (EMA) [16]. In return, the patent is protected for an additional 6-month period via a patent extension, for drugs used in orphan diseases from 10 to 12 years and medical products must be authorized in all EU member states. These rules apply for new medicines and new pharmaceutical forms, new methods of administration of existing authorized products [17–19]. Although regulators have implemented these measures to increase the number of paediatric clinical trials and, consequently, marketed new medicines for children, the impact on the number of clinical trials performed remains modest and new drugs authorized for the paediatric population are not accruing [20].

The pharmaceutical companies tend to initiate paediatric trials only if a sufficient return on investment is likely. As a consequence, the pharmaceutical industry will most likely focus on a very limited number of potentially profitable products [12]. From this perspective, non-commercial clinical trials represent a crucial tool for developing and updating the guidelines for clinical practice. Although NICTs usually provide little direct potential economic benefit as newly marketed drugs, the added value of paediatric NICTs lies in a high scientific benefit with transmission to clinical practice, appearing e.g. at the level of updated, optimized guidelines [21].

Generally, NICTs (also named academic) are focused on patient approach and an attempt to answer relevant questions from clinical practice. These trials include testing of currently authorized drugs for adults but in the paediatric population, comparisons between effectiveness and safety trials, or providing evidence of novel indications for registered drugs, including rare diseases. NICTs could also facilitate the accessibility of a new treatment in paediatric patients. Besides drugs, other interventions such as medical devices, nutrition, behaviour or therapeutic, diagnostic and surgical procedures are included in NICTs. Above all, NICTs provide robust evidence to improve therapeutic guidelines, support clinical decisions and enable policy makers to make sustainable policy decisions on public health [22].

In this regard, it is possible to define the level of quality of applied biomedicine research in the country using NICTs as a measurement, not only the number of ongoing studies but also the quality of the results publications from the finished trials. The main objective of this work is to identify and analyse the main trends and shortcomings, along with their impact on national performance in paediatric NICTs.

## Methods

In accordance with the main goal of this work—investigating paediatric NICTs to discover exactly how these types of trials are currently being conducted. We identified all paediatric NICTs which were registered between the dates 01/01/2004 to 31/12/2017 from recruitment sites in the Czech Republic.

This work represents a unique overview of the current landscape of paediatric NICTs in the Czech Republic.

### Search Methodology

Data related to all non-commercial interventional clinical trials from 4 international clinical trials databases were collected. It was comprised of information about trials that had been registered between the dates 01/01/2004 to 31/12/2017and marked as “Non-commercial” and “Interventional” trials.

The selection of the databases used for the search was based on previous research undertaken by Madeira et al. [23], namely:

- ClinicalTrials.gov
- The European Union Clinical Trials Register (EUCTR)
- BioMed Central International Standard Randomized Controlled Trial Number Registry (ISCTRN)
- Australia and New Zealand Clinical Trials Registry (ANZCTR)

The search strategy was different in each of the databases noted above, therefore the input of search keywords was varied. The detailed search methodology is specified in Table 1. The database searches covered the period from 01/01/2004 until 31/12/2017. If possible, the terms “Non-commercial”, “Interventional” trial and the name of the country “Czech Republic” were used in the general search field in combination with an individual inspection.

**Table 1.** The Detailed Search Methodology.

Keys Terms	Display in the Search Field as			
	ClinicalTrials.gov	EUCTR	ISCTRN	ANZCTR
Interventional	Trial type: interventional	Not applicable (NA) This database contains only information on interventional clinical trials	NA	Trial type: interventional
Non-commercial	Additional Criteria Funder Type: National Institutes of Health, All others, etc	General search field: Non-commercial	NA	Primary sponsor type: Government body, Hospital, etc. besides Commercial sector/industry)
Country	Locations: Country	Select Country	Countries of recruitment:	Countries of recruitment
Timeframe from 01/01/2004 until 31/12/2017	Additional Criteria: Start date from–to	Select Date Range: from–to	Date applied: from–to	Trial start date: from–to
Population	Child (birth–17)	Adolescent; Children; In utero; Infant and Toddler; Newborn; Preterm new born infants; Under 18	Neonate; Child	Child (under 18 years)
Link	www.clinicaltrials.gov	www.clinicaltrialsregister.eu	https://www.isrctn.com/	www.anzctr.org.au

*EUCTR* The European Union Clinical Trials Register, *ISCTRN* BioMed Central International Standard Randomized Controlled Trial Number Registry, *ANZCTR* Australia and New Zealand Clinical Trials Registry.

The search of the EU Clinical Trials Register (www.clinicaltrialsregister.eu) was performed using the time interval between two dates (from 01/01/2004 until 31/12/2017) and with the terms "CZECH REPUBLIC and NON-COMMERCIAL" in the general search field.

At ClinicalTrials.gov it was not possible to distinguish between commercial and non-commercial trials therefore an advanced search was performed. The trials were extracted by selecting the options “Interventional”, the limited time period, the name of the country and the applied filter for the funder type: NIH (U.S. National Institutes of Health), Other U.S. Federal agency, All others (individuals, universities, organizations) and Industry.

At ANZCTR an advanced search was performed by selecting the terms “Interventional”, the name of the county and the referred timeframe in the relevant fields.

The ISCTRN registry is associated with the BiomedCentral database. In this database it was not possible to refine the search except by selecting the time period and the name of the country.

### Extraction and Complementary Information

The following information was manually extracted from the registries for each of the NICTs found and is grouped by clinical specialties within each database. The data elements of interest were: Trial Identification number (Trial ID)—main and secondary, population, recruitment status, sponsor name and country, trial phase (when applicable), intervention, purpose, therapeutic area, design characteristics, type of funding and, publications of the completed trials.

Finally, manual review was used to confirm the compliance with the inclusion and exclusion parameters.

Exclusion parameters were:

- Industry sponsored trials
- Starting before 01/01/2014
- Participant recruitment beyond the Czech Republic
- Non-intervention trials

To categorize the non-commercial sponsors the following categories were created: Disease-Specific Organization (Disease associations or research institutes dedicated to a specific therapeutic area), Foundation, Hospital, Others (e.g. Individual investigators acting as sponsors), University and Research Institutes (non-specific therapeutic area).

NICTs can be funded by one or more sources, e.g. a secondary sponsor or collaborator. All these sources of funding found in the searches have been assigned to the predefined categories:

- Not-for-Profit organization (such as public institutions, funding agencies, disease-specific organizations)
- For Profit Organizations, when a private company is identified (Industry)
- Both, when the funding is provided from industry and another not-for-profit organization
- Not clear, when the information is not provided in any registry.

Because clinicaltrials.gov does not require funding information, the funding source was delivered on the basis of the “collaborator” fields and the secondary IDs in some cases. The support of funding agencies was perceived through secondary IDs where the code of the grant agreement was added in some cases.

The field “Source(s) of Monetary Support” in the WHO-ICTRP database (World Health Organization's International Clinical Trials Registry Platform) is fed by the information from the field “Sponsor and collaborators” from ClinicalTrials.gov. Therefore, when the funder was unclear on ClinicalTrials.gov and only the sponsor was listed under ‘sponsor’, those mentioned under WHO-ICTRP in the section “Source(s) of Monetary Support”, were considered as funders also.

### Identification of Duplicate Records

Some NICTs are registered in more than one register. A search allowing the identification of replicated trials was performed with the use of the excel function to identify duplicates comparing titles, sponsor and the main and secondary trial identification number public search function. The duplicates were excluded and not considered for the total number.

### Searching Publications of Completed NICTs

To determine whether trials had published manuscripts in scientific literature, the characteristics of the trial (such as the titles, ID, names of the investigators and acronyms associated with the trial) were used to search both the PubMed database and Google.

Searches of results reporting were restricted to completed trials until the end of 2017.

Additionally, manual review was undertaken to complete information about the funding sources of all published trials to identify any other relevant information that may have been missed by registry searches.

### Identification of Paediatric NICTs

A later step was focused on selecting trials that involved the paediatric population. Trials with young participants were identified by manual review according to the trial characteristic “population” in our database. The records were considered acceptable if the participant age range was specified (0–18 years) (for more details, see Table 1).

Finally, around 40% of the data were checked by an independent expert for appropriate content and consistency, ensuring that entries were relevant and correct. The evaluation of the results was restricted to paediatric trials only. The descriptive statistic has been used.

## Results

### Identification of Non-commercial Paediatric Trials

A total of 3496 interventional trials were identified in the four international clinical trials databases and were screened individually to isolate those that had a non-commercial sponsor, mention the Czech Republic as a country of the sponsor or recruiting site, involved the paediatric population and started between the dates of 01/01/2004 to 31/12/2017.

**Figure 1.**
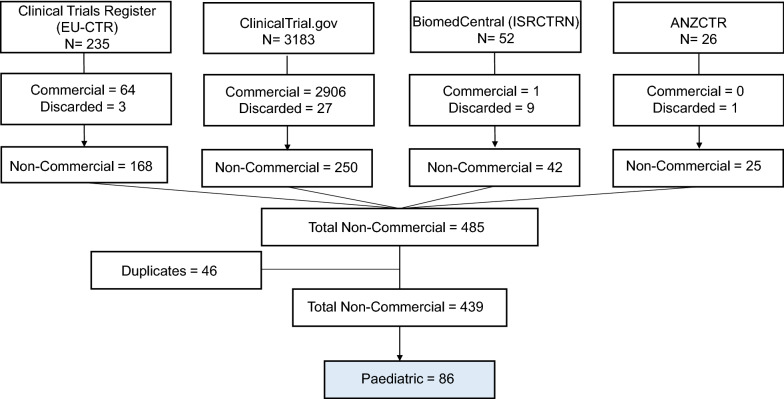
Flowchart Depicting the Systematic Search of Paediatric NICTs in Each Database. The Search was Performed on Four International Clinical Trials Registries (EUCTR, Clinicaltrials.gov, ISRCTN and ANZCTR). Clinical Trials Starting from 01/01/2004 until 31/12/2017 were Identified in Each of the Database Registries. After Discarding Commercial Trials, All Remaining CTs were Gathered into One Excel Sheet. Duplicate CTs were also Discarded. Paediatric CTs were Identified via Manual REVIEW According to the Trial Characteristic “Population”.

In Fig. 1 the number of registered trials found in each database is shown. From all the screened trials, 2971 were found to have a commercial sponsor. Although marked as non-commercial trials, 46 were recognized as duplicates and the other 40 were also discarded because they did not meet the inclusion criteria (the start of the trial was beyond the time period or they were only observational). Those all were removed and not considered in the total number.

Removing these from the original total of 3496, the search identified 439 unique NICTs with non-commercial sponsors conducted in the Czech Republic. When stratifying these trials by enrolment age, 86 trials were found to involve the paediatric population.

**Figure 2.**
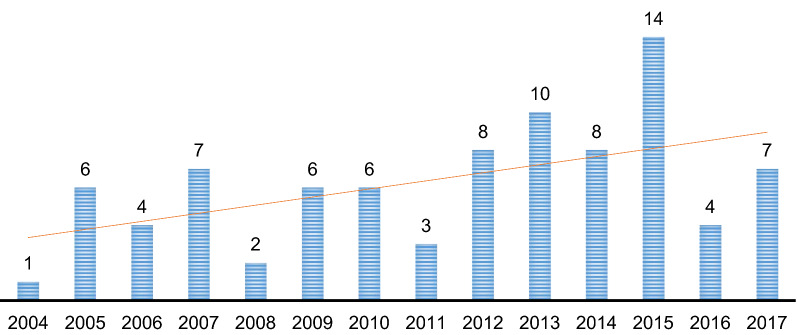
The Number of Paediatric NICTs Started in Each Year During the Time Period 01/01/2004 to 31/12/2017 (*N* = 86).

Within the referred timeframe, between the dates of 01/01/2004 to 31/12/2017, a slightly increasing trend with some upward or downward deflection can be observed. The greatest number of trials were started in the years 2013 and 2015. By contrast, in the year 2004 only one trial was started and a similar situation can be seen in the year 2008 (*N* = 2) and 2011 (*N* = 3). The sharpest rise came in the year 2005, when the number increased to 6, which was largely attributable to the implementation of the ICMJE (International Committee of Medical Journal Editors [21]) policy. In the remaining years of the period the number of the trials was stable (Fig. 2).

As shown in Table 2, hospitals (*N* = 35), universities (*N* = 33) and disease-specific organizations (*N* = 14) are the most frequent sponsors of non-commercial paediatric trials.

**Table 2.** The Number of Paediatric NICTs According to the Sponsor (*N* = 86).

Sponsor	Total Number
Hospital	35
University	33
Disease Specific Organization	14
Research Institute	3
Foundation	1

The focus of paediatric non-commercial trials is most commonly oncology (*N* = 29), cardiology (*N* = 7) and metabolic disease (*N* = 5), in the centre of this spectrum are haematology, neurology, infectious disease and gastroenterology (*N* = 4). Less commonly involved are therapeutic areas such as musculoskeletal system disease (*N* = 1) and ophthalmology (*N* = 1; Table 3).

**Table 3.** The Number of Paediatric NICTs According to the Therapeutic Area (*N* = 86).

Therapeutic Area	Total Number
Oncology	29
Cardiology	7
Metabolic Diseases	5
Otolaryngologists	4
Gastroenterology	4
Immunology	4
Infectious Diseases	4
Neonatology	4
Neurology	4
Haematology	4
Others	3
Anaesthesiology	3
Traumatology	3
Pain	2
Dermatology	2
Respiratory Diseases	2
Ophthalmology	1
Musculoskeletal System Diseases	1

The majority of the trials (*N* = 60) were focused on medicinal products versus other interventions as a procedure (*N* = 6), surgery (*N* = 5), diagnostic method (*N* = 4) or medical device (*N* = 4; Table 4). Most trials testing medicinal products are in phase III (*N* = 22), followed by phase II and IV (*N* = 60; Table 5).

**Table 4.** The Number of Paediatric NICTs According to the Type of Intervention (*N* = 86).

Type of Intervention	Total Number
Medicinal product	60
Procedure	6
Surgery	5
Behaviour	4
Medical device	4
Diagnostic method	4
Nutrition	3

**Table 5.** The Number of Paediatric NICTs with Medicinal Products Divided According to the Type of Phase (*N* = 60)

Type of Phase	Total Number
Bioequivalence	1
I	1
I/II	3
II	11
II/III	4
III	22
III/IV	1
IV	7
Not specified	10

The majority of the trials had an international sponsor (*N* = 57; Table 6A). A small percentage of the paediatric trials with national sponsors were conducted as international (*N* = 3, 10%; Table 6B).

**Table 6.** Distribution of NICTs According to the Origin of Sponsor (A) International vs National and Involvement of Other Countries in CTs with National Sponsors (B) National vs International Trial

(A) Origin of Sponsor	Total number
International sponsor paediatric NICTs	57
National sponsor paediatric NICTs	29

(B) Type of Trial with National Sponsor	Total number
National trial	26
International trial	3

In total, 48 trials (56%) were monocentric and 23 (27%) trials were conducted as a multicentre (Table 7A). Most of the trials were randomized (*N* = 53, 62%; Table 7B).

**Table 7.** The Number of Paediatric NICTs According to Type of Clinical Trial (A) Monocentric vs Multicentre and (B) Randomized vs Non-randomized (*N* = 86).

(A) Type of Clinical Trial	Total Number
Monocentric	48
Multicentre	23
Not specified	15

(B) Type of Clinical Trial	Total Number
Randomized	53
Non-randomized	21
Not specified	12

**Figure 3.**
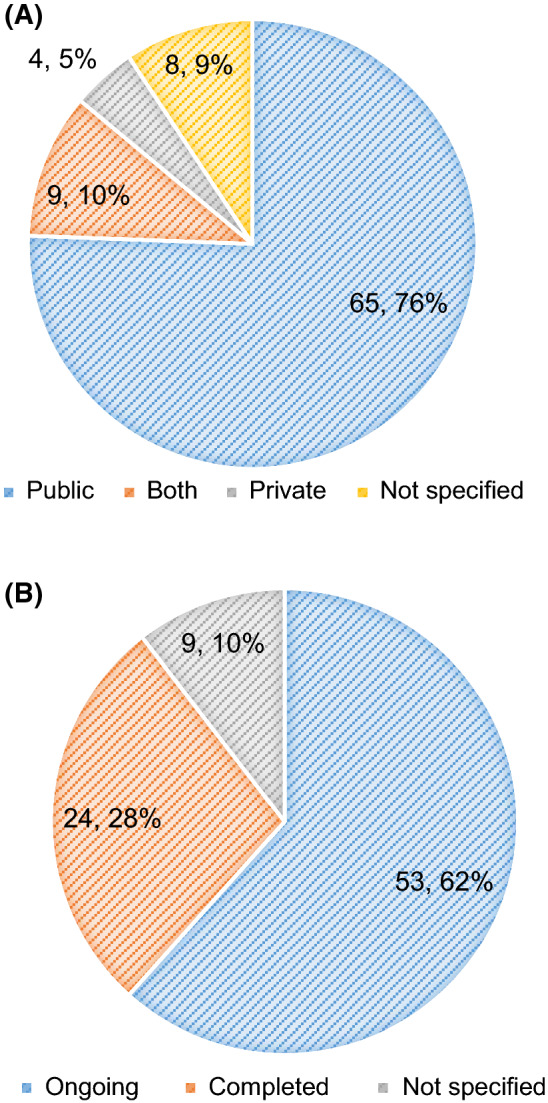
**a** The Percentage and Number of Paediatric NICTs According to the Type of Funder (*N* = 86). **b** The Percentage and Number of the Paediatric NICTs According to the Status (*N* = 86).

The analysis of the funding sources showed that more than two-thirds of trials were funded by a public organization (*N* = 65, 76%); generally, grant agencies, government bodies or other non-commercial organizations. Of those trials, a total of 12 received financial support from international funding agencies such as the European Commission and 5 from national funding agencies such as the Internal Grant Agency of the Czech Ministry of Health. Industry was identified as the funding source for 4 trials (5%), while 9 trials (10%) were funded by both private and public organizations. No information regarding the funder was reported for 9% of the trials (Fig. 3A).

At the moment, more than half of the trials (*N* = 53, 62%) are active and are recruiting patients at the time of writing. The total number of completed paediatric trials was 24 (28%); only 12 of them have already published the results, which amount to 50% (Fig. 3B). Most of the published trials were focused on the testing of medicinal products (*N* = 7) and were published in journals with an impact factor lower than 5 (*N* = 6). In 83% (*N* = 10) of the published trials the funding source was from public organizations (Table 8).

**Table 8.** Characteristic of the Published Paediatric NICTs (*N* = 12).

Intervention		Impact Factor		Funders	
Behaviour	2	0 to 5	6	Non-profit organization	10
Device	1	6 to 20	5	Both	2
Medicinal Product	7	21 and higher	1		
Surgery	2				

## Discussion

For the first time, this work provides a unique overview of the facts about paediatric investigator-initiated clinical trials in the CR between 01/01/2004 and 31/12/2017. This period includes the time after 2004, the year of the International Committee of Medical Journal Editors’ implementation (ICMJE), policy requiring the registration of clinical trials as a prerequisite for consideration for publication [21].

The work examined the data collected from four clinical trial public databases to describe the current status of paediatric NICTs in the CR with the aim of defining trends and shortcomings, knowledge of which would help to increase the level and quality of independent non-commercial clinical research in the CR. The type of sponsor, intervention, phase, therapeutic area, design characteristics, and type of funding were reviewed and analysed.

In practice, conducting NICTs is extremely time-consuming and requires good logistics and management services. Limited financial and human resources hamper the organization of NICTs which have to comply with the same regulatory requirements as big pharmaceutical companies investigating a novel therapy [24]. The limitation of funding from national grants is the cost eligibility. Only national costs are eligible, which means to only national trial can be conducted. However, to get a powerful sample, you need multinational trials. For university hospital physicians with an enormous workload of teaching and ensuring medical care, preparing, initiating and enacting a multinational clinical trial is challenging [25]. The competition involved in obtaining financial resources from a European grant takes great effort, time and expertise. Physicians are not generally well trained in the application process for the grant funding. Therefore, the support of specialists in this area is crucial [26]. Furthermore, the long delay between application and funding all the way to approval can lead to the failure of even starting NICTs.

The limitation of these funds is usually a short timeframe for patient recruitment and the fact that they generally do not allow subcontracting with for-profit Clinical Research Organizations (CROs) and involve other centres in different European countries.

In some cases, NICTs can also be funded by other not-for-profit organizations such as patient associations, governmental organizations and foundations [27]. Private sources of funding for NICTs are increasing over recent years. Participation of private companies may also be in the form of drug product supply, financial resources or both. With this type of collaboration between pharmaceutical companies and an individual non-commercial investigator, there may be a risk that the company struggles to persuade the investigator to work on defined priorities rather than their own research aims [28]. In the face of this, these trials should not be truly considered in the sense of independent non-commercial research.

Thus, the continuous funding and commitment of governments is an important step, as is mentioned in the upcoming Clinical Trial Regulation (Regulation (EU) No 536/2014)—*In order to maximize the valuable contribution of such non-commercial sponsors and to further stimulate their research but without compromising the quality of clinical trials, measures should be taken by Member States to encourage clinical trials conducted by those sponsors* [29].

During the last decade several EU-level guidelines came into force requiring the conduct of more paediatric clinical trials in order to force the development and accessibility of medical products for children [30, 31]. In line with this there has been a gradual shift to the current consensus that children deserve the same level of healthcare as any other age group [32]. It was acknowledged that this initiative had a positive impact and led to an increase in the number of non-commercial trials [33, 34]. The implementation of Directive 2001/20/EC certainly improves the quality of clinical trials and assures the safety and well-being of trial subjects [35]. Nevertheless, at the same time, performing an NICT according to the new requirements is nearly impossible for clinicians and non-commercial researchers without cooperating with expensive specialized experts, such as project managers, statisticians, data managers, pharmacists and monitors [36]. This area also presents a lot of challenges mainly due to differences in national law and practice; without industry sponsorships, conducting paediatric NICTs seems almost unthinkable. Indeed, the investigators spend a lot of time and resources on planning and preparing study documentation in order to successfully submit to the relevant regulatory authorities and ethics committees.

Also, Fig. 2 supports the conclusion that regulatory policy has had a slight effect on paediatric trials practice in the CR. The number of paediatric NICTs conducted in CR grew between 2004 and 2017. On the other hand, these NICTs seem to have been registered in order to comply with ICMJE policy [21]. In 2016, there was a significant decrease in the number of NICTs, which could be explained as an impact of the termination of the Internal Grant Agencies under the Ministry of Health in the CR in 2014 and the establishment of the new Agency for Health Research a year later [37]. Researchers had limited opportunities to gain resources to support NICTs, which could have reduced the number of registered NICTs.

Over half of the trials have international sponsors, which is a good indicator of CR’s attractiveness. Despite this, only a small number of NICTs initiated by a national sponsor were conducted as multinational and multicentric [38]. This can likely be explained by the condition of national grants that do not allow the provision of financial support to foreign partners. By contrast, Belgium and Denmark, for example, have the different rules [39, 40]. Public national funds in those countries can also be used for the involvement of foreign partners. This fact fosters the implementation of multinational, multicentre trials and also boosts patient recruitment and often leads to an improved collaboration.

Furthermore, there are other impediments when conducting multinational trials including regulatory fragmentation in participating countries’ centres (such as an informed consent/assent, ethical review, pharmacovigilance, data monitoring, language and insurance) [41].

The analysis of therapeutic areas shows that NICTs cover a wide range, with oncology and cardiology at the forefront. Next in line are metabolic diseases, haematology, neurology, infectious diseases/immunology and gastroenterology, but no particular area dominates. Generally, this is a good sign as it demonstrates that paediatric research is covering a wide range of diseases. Most paediatric NICTs are randomized and most trials test drugs in Phase III which corresponds to the fact that non-commercial trials typically test already licensed drugs for a new indication, different route of administration, or new dose/dosage form.

Overall, paediatric research has gained a boost in all of Europe as is shown in the EMA report. The proportion of clinical trials in the European clinical trial database EudraCT that includes children has increased by 50% in 2007–2016 from 8.25 to 12.4% [30].

Indeed, CR is below the European average in regards to conducting non-commercial clinical trials. Only 4% of all registered clinical trials are initiated by investigators without industry sponsorship, while the European average is 17% [42].

The registration of trials and provision of basic information about a sponsor, protocol and results in a publicly accessible registry is the first step to promoting transparency in clinical trials research and it is also one of ethical principles of the Declaration of Helsinki [43]. The primary responsibility for registration and providing data lands with the sponsors, principal investigators and in some instances other persons/organizations authorized by the sponsor. The provided data should be current and complete, although in some cases information was not available in the screened registries. This is probably due to the fact that completion of some items is now optional. Future improvement could be to make full completion of the individual trial in the registry, before its approval, mandatory, including the fields referring to a funder and ownership of the results. According to the presented results, most of the paediatric NICTs in CR (76%, *N* = 65) are funded by non-commercial organizations, 15% of the NICTs received support from the private sector. This support could be financial, the supply of medical products/devices or both; when the trial is funded or supported by a private company it is often not clear who will be the owner of the obtained data. If the owner is a private company, the trial should not be considered as independent non-commercial research. According to the screened registries, it is not possible to find out who is ownership of the results. Nowadays, some journals have introduced a requirement of the full disclosure of funders [23].

From the total number of completed non-commercial trials, (*N* = 24) only half of them have already published the results, despite the duty stated in the Declaration of Helsinki. Additionally, in the case of clinical trials on paediatric subjects the results should be publicly available 6 months after the end of the trial (generally the obligation for public results of clinical trials is one year, [17, 43]). This corresponds to the cohort study published in The Lancet where compliance with legal requirement to report clinical trial results on ClinicalTrials.gov was followed. Only 1722 trials from the 4209 registered in our particular timeframe on ClinicalTrials.gov reported results within a 1-year period [44].

## Conclusion

Generally, non-commercial clinical trials are the basis of a country's clinical research strength [45, 46]. They have a crucial importance for healthcare provision, as they answer questions which are highly relevant but often disregarded by the pharmaceutical industry, especially in the case of the paediatric population due to its very low profitability [47]. However, those trials still represent a relatively small percentage compared to the commercial ones [48].

One potential way to support paediatric clinical trials could be via European infrastructures such as the ECRIN [49], particularly the PedCRIN project which is focused on developing the capacity for the management of multinational paediatric non-commercial clinical trials [50]. At this time CR is a full member of the ECRIN infrastructure and a national network named Czech Clinical Research Infrastructure Network (CZECRIN) was established at the end of 2014 [51, 52]. The aim of CZECRIN is to create a sustainable research environment and help researchers with the planning and conducting of NICTs [53]. The offered support encompasses, for instance, submission procedure to regulatory authorities, trial management, pharmacovigilance, monitoring or data management. CZECRIN has established a network of Clinical Trial Centres at Czech medical faculties/university hospitals with the aim to deliver these scientific services across this network [51].

One of the most pressing issues is public funds. There is a need to simplify and refine the financing rules for the future which include all the stakeholders. From the healthcare providers, to the government, grant agencies, research institutions and industry, we need to make international costs more affordable, where the sponsor comes from the CR but NICTs must be multinational for the aforementioned reasons. New concepts of funding should be set up, for example specific grants of the European Union or their member states. Furthermore, pharmaceutical companies and all stakeholders involved in paediatric clinical trials must realize that the small financial contributions of the past are not sufficient any more to perform high quality paediatric IITs according to Directive 2001/20/EC [35].

Another important step in order to follow future evaluations and comparison of NICTs performance is improving the accuracy of clinical trials’ registrations. A legal mandate for trial registration, not just for medical products, including disclosure of the funder and respective ownership of the results could also have to impact on the transparency of clinical research. A comparison of other European countries could be a useful scientific indicator; however, it is difficult because publications allocated to this topic are not commonly available.

## Electronic Supplementary Material

Below is the link to the electronic supplementary material.

Supplementary file1 (XLSX 148 kb)

## Data Availability

The datasets collected and analysed during the searches are available as supplemental file.

## Abbreviations

NICTs: Non-commercial Interventional Clinical Trials
ANZCTR: Australia and New Zealand Clinical Trials Registry
CR: Czech Republic
CROs: Clinical Research Organizations
CTs: Clinical Trials
CZECRIN: Czech Clinical Research Infrastructure Network
ECRIN: European Clinical Research Infrastructure Network
EMA: European Medicines Agency
EUCTR: European Union Clinical Trials Register
ICMJ: International Committee of Medical Journal Editors
ID: Identification Number
ISCTRN: International Standard Randomized Controlled Trial Number Registry
NIH: National Institutes of Health
PedCRIN: Paediatric Clinical Research Infrastructure Network
WHO-ICTRP: World Health Organization's International Clinical Trials Registry Platform
